# Targeting of colony-stimulating factor 1 receptor (CSF1R) in the CLL microenvironment yields antineoplastic activity in primary patient samples

**DOI:** 10.18632/oncotarget.25191

**Published:** 2018-05-15

**Authors:** David K. Edwards V, David Tyler Sweeney, Hibery Ho, Christopher A. Eide, Angela Rofelty, Anupriya Agarwal, Selina Qiuying Liu, Alexey V. Danilov, Patrice Lee, David Chantry, Shannon K. McWeeney, Brian J. Druker, Jeffrey W. Tyner, Stephen E. Spurgeon, Marc M. Loriaux

**Affiliations:** ^1^ Department of Cell, Developmental and Cancer Biology, Oregon Health & Science University, Portland, OR, USA; ^2^ Division of Hematology & Medical Oncology, Oregon Health & Science University Knight Cancer Institute, Portland, OR, USA; ^3^ Array BioPharma, Boulder, CO, USA; ^4^ Department of Bioinformatics and Computational Biology, Oregon Health & Science University, Portland, OR, USA; ^5^ Howard Hughes Medical Institute, Oregon Health & Science University, Knight Cancer Institute, Portland, OR, USA

**Keywords:** colony-stimulating factor 1 receptor, chronic lymphocytic leukemia, tumor-associated macrophages, tumor microenvironment, small-molecule inhibitors

## Abstract

In many malignancies, the tumor microenvironment includes CSF1R-expressing supportive monocyte/macrophages that promote tumor cell survival. For chronic lymphocytic leukemia (CLL), these supportive monocyte/macrophages are known as nurse-like cells (NLCs), although the potential effectiveness of selective small-molecule inhibitors of CSF1R against CLL is understudied. Here, we demonstrate the preclinical activity of two inhibitors of CSF1R, GW-2580 and ARRY-382, in primary CLL patient samples. We observed at least 25% of CLL samples showed sub-micromolar sensitivity to CSF1R inhibitors. This sensitivity was observed in samples with varying genetic and clinical backgrounds, although higher white cell count and monocyte cell percentage was associated with increased sensitivity. Depleting CD14-expressing monocytes preferentially decreased viability in samples sensitive to CSF1R inhibitors, and treating samples with CSF1R inhibitors eliminated the presence of NLCs in long-term culture conditions. These results indicate that CSF1R small-molecule inhibitors target CD14-expressing monocytes in the CLL microenvironment, thereby depriving leukemia cells of extrinsic support signals. In addition, significant synergy was observed combining CSF1R inhibitors with idelalisib or ibrutinib, two current CLL therapies that disrupt tumor cell intrinsic B-cell receptor signaling. These findings support the concept of simultaneously targeting supportive NLCs and CLL cells and demonstrate the potential clinical utility of this combination.

## INTRODUCTION

Chronic lymphocytic leukemia (CLL) is the most common leukemia in the Western world, with nearly 19,000 new cases reported annually in the United States [1]. The disease is characterized by an accumulation of small mature B-lymphocytes in the lymph nodes, bone marrow and peripheral blood. CLL is predominantly an indolent disease; however, around 25% of patients progress rapidly [2].

Therapy is generally reserved for patients with symptomatic disease and, until recently, has largely relied on chemo-immunotherapy combination regimens. Introduction of novel targeted therapies that inhibit B-cell receptor (BCR) signaling—such as ibrutinib, a Bruton’s tyrosine kinase (BTK) inhibitor, and idelalisib, a phosphatidylinositol-3-kinase delta isoform specific (PI3kδ) inhibitor—have dramatically improved patient outcomes and treatment options [3]. However, CLL remains incurable with these classical treatments, and most patients succumb to the disease or its complications.

One significant barrier to treatment is the contribution of the tumor microenvironment, which has been shown to be critical for cancer cell growth and survival. Tumor-associated macrophages (TAMs) have been shown to provide microenvironmental support that maintains tumor cell viability and proliferation in a variety of solid tumor types [4]. These TAMs have protean pro-survival effects including increased angiogenesis, tumor cell invasion, metastasis, and inhibition of immune-mediated anti-tumor responses [5]. TAMs have also been isolated from the peripheral blood, spleen, and lymph nodes in CLL patients where they have shown to be essential for CLL cell survival in the tumor microenvironment [6].

In this setting, these TAMs, which are known as nurse-like cells (NLCs) or lymphoid-associated macrophages (LAMs), share a similar gene expression profile to TAMs derived from other tumor types [7]. Specifically, these NLCs are derived from CD14-positive monocytes and, in the presence of CLL cells, differentiate into abnormal macrophages [8-11], which promote CLL cell survival [8]. This has particular clinical relevance given the finding that elevated peripheral blood monocyte count at the time of CLL diagnosis is associated with inferior outcomes [12].

In solid tumors, TAM function has been shown to depend on the receptor tyrosine kinase CSF1R [13]. CSF1R, also known as cFMS and M-CSFR, is a member of the type III receptor tyrosine kinase family and is activated by binding of its ligands CSF-1 (MCSF) or IL-34 [14-16]. CSF1R is predominantly expressed on monocytes and tissue macrophages [17, 18] and is required for proliferation [19], differentiation [20], and chemotaxis [21], all functions critical to TAM activity.

Recent studies suggest an important potential role for targeting CSF1R in CLL. In mice, depletion or targeting of TAMs has been associated with reduction in leukemic burden *via* reprogramming of the tumor microenvironment [22, 23]. Furthermore, using patient samples, neutralization or inhibition of CSF1R has been shown to inhibit NLC formation and decrease CLL cell viability, a finding mimicked by NLC depletion [24]. Given the role of NLCs in CLL as well as possible therapeutic implications, we evaluated the impact of CSF1R inhibition using highly selective small-molecule inhibitors across a broad spectrum of primary CLL samples.

## RESULTS

### CLL patient specimens are sensitive to CSF1R-specific small-molecule inhibitors

**Figure 1:**
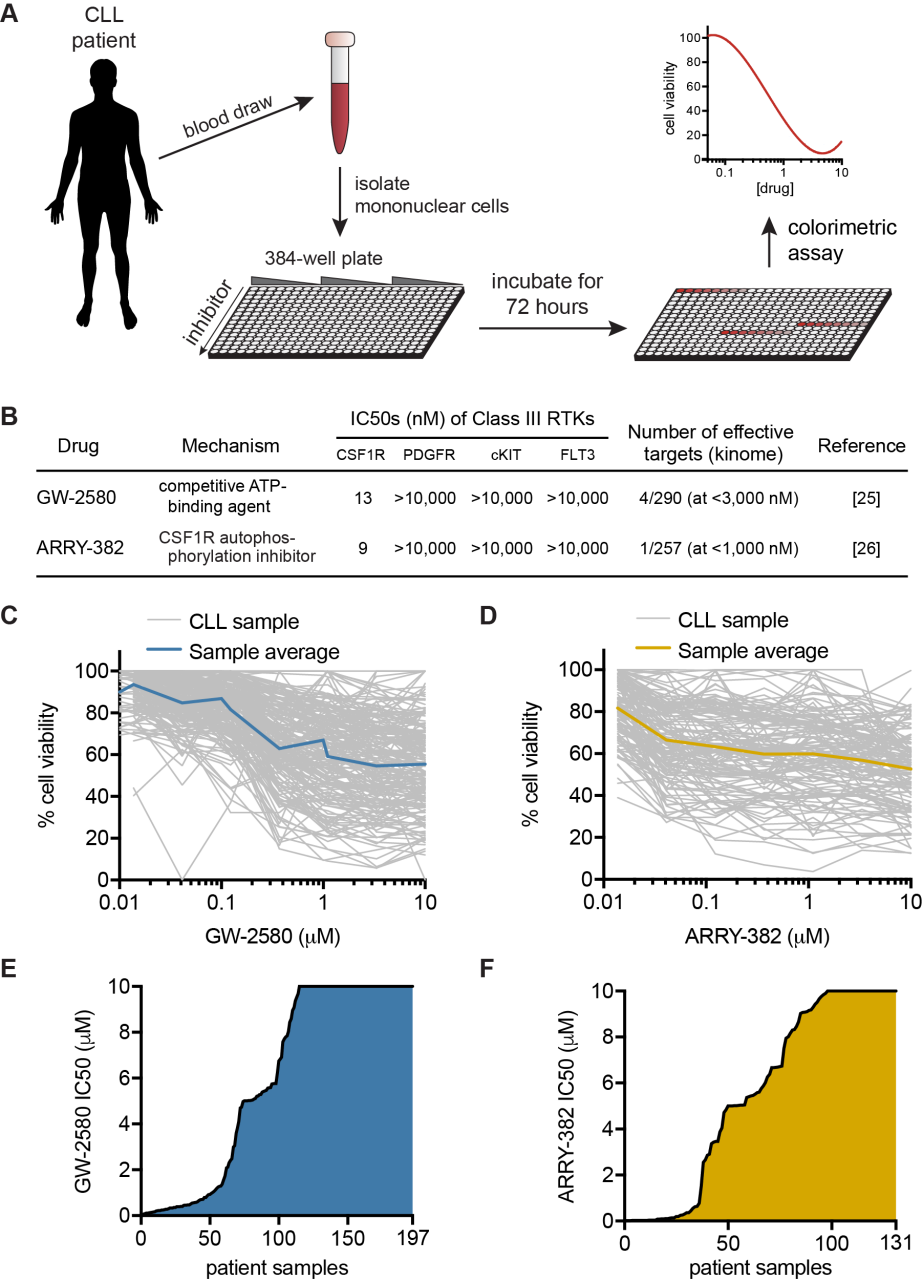
*Ex vivo* inhibitor screening reveals CSF1R sensitivity in CLL patient specimens **A.** Mononuclear cells isolated from peripheral blood or bone marrow of CLL patients were added to 384-well plates containing dose-escalating concentrations of small-molecule inhibitors. Following incubation for 72 hours, the relative number of remaining viable mononuclear cells was evaluated by subjecting cells to a colorimetric cell viability assay. **B.** GW-2580 and ARRY-382 are highly specific small-molecule inhibitors of CSF1R (and not other class III receptor tyrosine kinases). **C.**-**D.** CLL primary patient specimens were exposed to **C.** GW-2580 and **D.** ARRY-382, as described in **A.**, and dose-response curves for each specimen were included along with an average dose-response curve for all specimens. **E.**-**F.** Waterfall plot of the IC50 values for each patient specimen after exposure to **E.** GW-2580 and **F.** ARRY-382. The IC50 was calculated from the dose-response curve using a cubic logarithmic regression, and each specimen was positioned in order of increasing IC50.

We analyzed primary CLL samples using an *ex vivo* functional screen in which cells were exposed to dose-escalating concentrations of small-molecule inhibitors for 72 hours and then relative numbers of viable cells were assessed to generate dose-response curves (Figure 1A). The inhibitors tested included the highly selective CSF1R inhibitors GW-2580 (*n* = 197) (GlaxoSmithKline) and ARRY-382 (*n* = 131) (Array BioPharma), the latter of which has completed Phase I clinical testing. Both inhibitors exhibit a high degree of specificity for CSF1R across the kinome, including other class III receptor tyrosine kinases (Figure 1B) [25, 26]. We observed that a proportion of CLL specimens showed sensitivity to these selective CSF1R inhibitors, with 25.9% (51/197) and 27.5% (36/131) of specimens showing sub-micromolar IC50s (the concentration of inhibitor required to reduce viability to 50%) for GW-2580 and ARRY-382, respectively (Figure 1C-1D). We confirmed that increased exposure to CSF1R inhibitors induced apoptosis in patient sample cells *via* annexin V staining (Supplementary Figure 1).

**Figure 2:**
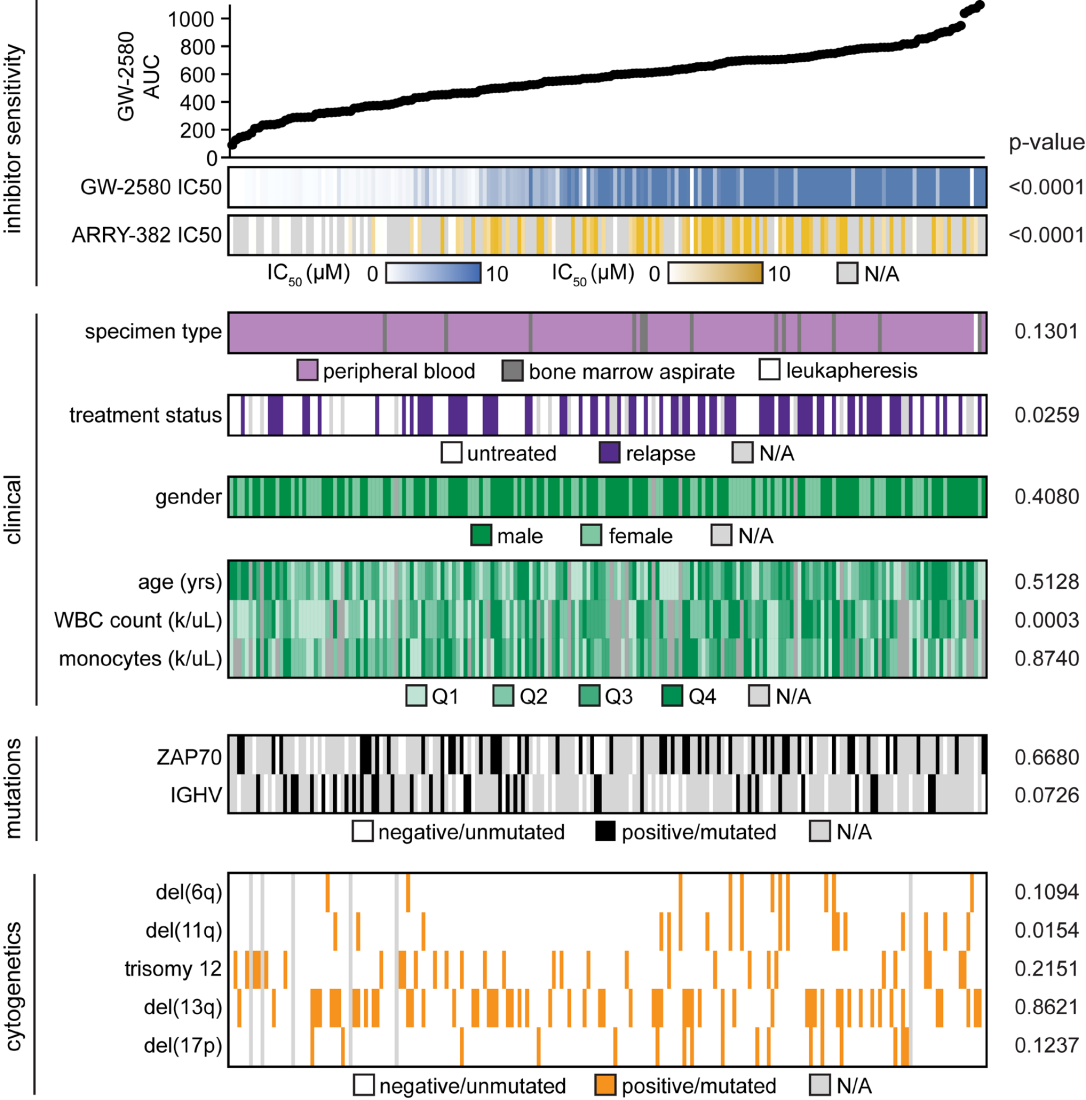
No genetic or clinical characteristic readily co-segregate with sensitivity to CSF1R inhibition in CLL patient samples The 197 CLL patient specimens that were evaluated by *ex vivo* inhibitor screening in Figure 1 were ordered by increasing AUC for GW-2580, which was calculated using a cubic logarithmic regression model. Various demographic, clinical, and genetic/cytogenetic characteristics of each patient were determined (the continuous variables are broken into quartiles) and each characteristic was evaluated for statistical significance (see Supplementary Figure 2 and Supplementary Tables 1-2).

Previous genomic analyses of CLL patients have identified no mutations in CSF1R [27, 28], nor is CSF1R significantly overexpressed in CLL compared to healthy monocytes. To identify a potential association with known clinical and biological characteristics, we evaluated these characteristics across the cohort of patient specimens that had been screened for CSF1R inhibitor sensitivity (Figures 2 and Supplementary Figure 1A; Supplementary Tables 1-2). For GW-2580 and ARRY-382, the IC50 and average area under the curve (AUC) were calculated for each patient specimen, and the specimens were organized by decreasing sensitivity to GW-2580. As expected, we observed a strong correlation between GW-2580 IC50 and GW-2580 AUC, and between GW-2580 AUC and ARRY-382 AUC (*p* < 0.0001; Supplementary Figure 2B-2C). We did not observe an association between specimen type (either from peripheral blood or bone marrow aspirate) and CSF1R inhibitor sensitivity (Supplementary Figure 2D).

We compared the CSF1R inhibitor sensitivity across CLL primary patient specimens with various clinical and genetic characteristics (Figure 2 and Supplementary Figure 1E-1P). Of the clinical characteristics, lower white blood cell (WBC) count is associated with sensitivity to CSF1R inhibitors. Furthermore, treatment status also significantly correlates with sensitivity to CSF1R inhibition, with relapsed patient specimens showing more resistance compared to specimens obtained from untreated patients. Additionally, of the cytogenetic abnormalities, deletion 11q is found more frequently in CSF1R-resistant patient specimens. However, none of these characteristics are uniformly enriched in the specimens that are sensitive to CSF1R inhibitors, suggesting that other mechanisms might be responsible for inhibitor response.

### CD14+ cell subpopulation expresses CSF1R and is associated with CSF1R inhibitor sensitivity

**Figure 3:**
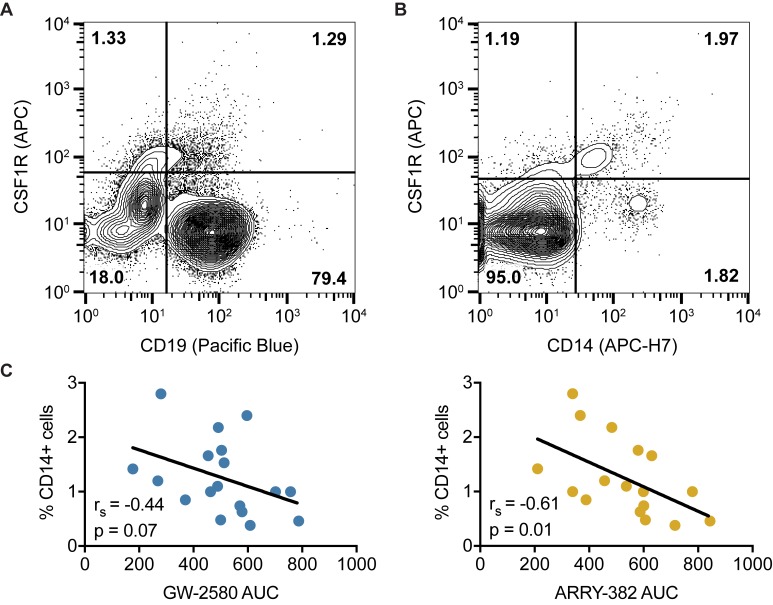
CSF1R is not found on CD19+ CLL cells but instead expressed on a CD14+ myeloid subpopulation **A.**-**B.** Mononuclear cells isolated from CLL patients were subjected to flow cytometry using antibodies specific for CSF1R and CD19, with CSF1R expression **A.** not observed in CD19+ lymphocytes (CLL cells) but **B.** observed in a subpopulation of CD14+ cells. **C.** Sensitivity to CSF1R inhibitors, as determined in Figures 1 and 2, was correlated with percentage of CD14-positive cells as determined in Figure 3B. Statistics were calculated using Spearman’s rank correlation.

Since no obvious characteristics of the CLL patient specimens readily co-segregated with CSF1R sensitivity, we examined the contribution of tumor-extrinsic factors. The tumor microenvironment has been shown to be critically important in the development of CLL and partly responsible for the ineffectiveness of modern chemotherapy regimens [29, 30]. We first assessed the profile of CSF1R expression across various cell populations in fresh CLL patient specimens using flow cytometry conducted by hematopathologists. Consistent with a tumor cell-extrinsic role of CSF1R, we did not find CSF1R expressed on CLL leukemic lymphocytes (CD5+/CD19+), but we did find it expressed on a subpopulation expressing CD14, a surface marker found predominantly on monocytes and macrophages (Figure 3A-3B).

These findings led to a hypothesis whereby CSF1R inhibitors act indirectly on CLL leukemic cells *via* direct inhibition of CSF1R-expressing monocytes, suggesting that the presence of varying levels of CSF1R-expressing monocytes may correlate with varying degrees of CSF1R inhibitor sensitivity. Therefore, we wanted to determine whether quantitative levels of CD14 expression correlate with CSF1R sensitivity. We compared the percentages of CD14-positive cells in patient samples, as measured by flow cytometry, to the AUC values for GW-2580 and ARRY-382. We found that a higher CD14-positive percentage of cells is associated with increased sensitivity to CSF1R inhibitors (*p* = 0.07 for GW-2580; *p* = 0.01 for ARRY-382) (Figure 3C). These results suggest that the CD14-positive subpopulation of cells is associated with CSF1R inhibitor sensitivity.

### CD14+ depletion in CLL patient samples decreases cell viability and eliminates CSF1R inhibitor sensitivity

**Figure 4:**
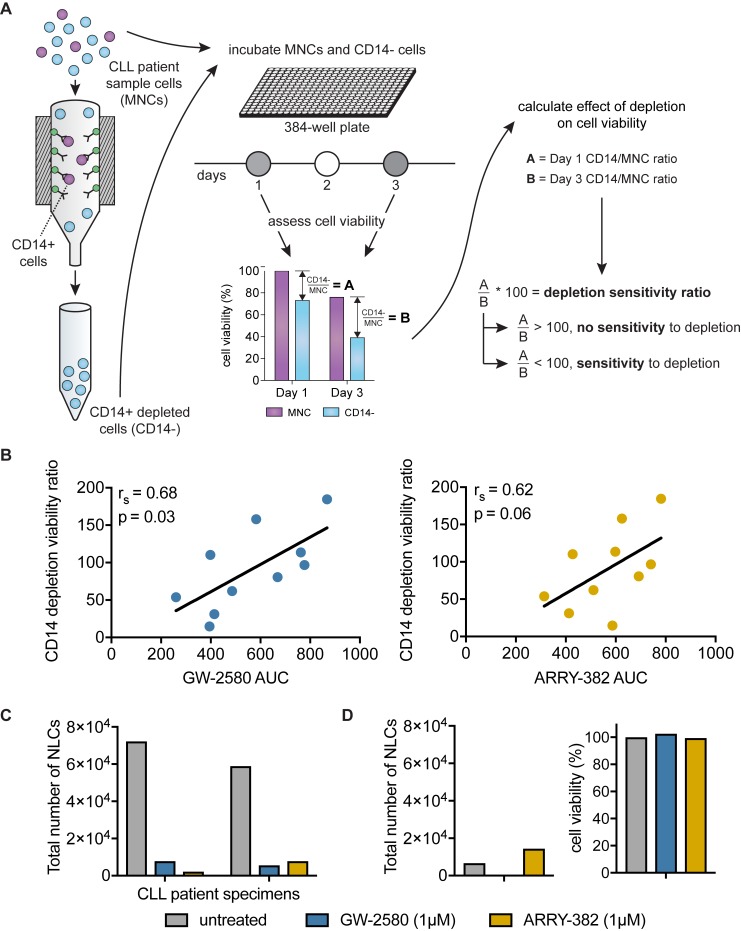
Sensitivity of CLL cells to CD14+ depletion correlates with sensitivity to CSF1R inhibitors **A.** CD14+ cells were depleted from patient specimens using magnetic cell-separation columns, and incubated in 384-well plates for 72 hours. The CD14+ depletion sensitivity ratio was calculated by comparing the relative remaining numbers of viable cells in depleted *versus* non-depleted conditions using a colorimetric assay. The ratio of cell viability readings in depleted to non-depleted cells at 72 hours was normalized to the same ratio at the start of the experiment to control for variance in cell input. **B.** The CD14+ depletion sensitivity ratio was generated for a panel of primary CLL patient samples as described in **A.**. This ratio was compared to GW-2580 and ARRY-382 AUCs. Statistics determined by Spearman’s rank correlation. **C.** Primary CLL patient samples cells were exposed to CSF1R inhibitors and were subjected to long-term culture conditions to produce nurse-like cells (NLCs). The number of NLCs was quantified using a hemocytometer. **D.** For one primary patient sample that did not produce NLCs, the addition of CSF1R inhibitors did not have a significant impact on cell viability compared to untreated control.

We next wanted to orthogonally validate that the impact of CSF1R inhibition on CLL cell viability is due to the presence (or, rather, the post-inhibitor depletion) of CSF1R-expressing CD14-positive cells. We therefore performed a CD14 antibody depletion experiment with magnetic column cell separation. After depleting CD14-positive cells, we incubated the remaining CD14-negative fraction (“depleted”) and whole mononuclear cells (“non-depleted”) from the same specimen for 72 hours and measured relative numbers of viable cells to compare the impacts of CD14+ depletion *versus* CSF1R inhibition on cell viability (Figure 4A). We confirmed that the depletion protocol itself has no significant effect on overall cell viability by measuring viability pre- and post-depletion (Supplementary Figure 3A).

To quantify the impact of CD14+ depletion, we generated a CD14+ depletion sensitivity ratio that expresses the number of viable cells in CD14+ depleted conditions relative to whole mononuclear cells after 3 days in culture (following normalization to the starting cell viability in order to correct for variance in cell input). A CD14+ depletion sensitivity ratio less than 100 indicates a deleterious effect of CD14+ depletion on the viability of the depleted cells relative to the viability of whole mononuclear cells from the same specimen. The CD14+ depletion sensitivity ratio was compared to CSF1R inhibitor sensitivity in non-depleted cells.

We observed a correlation between sensitivity to CD14+ depletion (sensitivity ratio less than 100) and sensitivity to CSF1R inhibition (*p* = 0.03 for GW-2580; *p* = 0.06 for ARRY-382) (Figure 4B). To confirm that this correlation was specific to CSF1R sensitivity and not more generally to overall drug sensitivity, we compared the CD14+ depletion sensitivity ratio against sensitivity to two small-molecule inhibitors that exhibit recurrent efficacy in CLL—ibrutinib, which targets Bruton’s tyrosine kinase (BTK), and idelalisib, which targets phosphoinositide 3-kinase delta (PI3Kδ). We confirmed that there is no association between CSF1R inhibitor sensitivity and sensitivity to ibrutinib or idelalisib (Supplementary Figure 3B-3C). In addition, we observed no correlation between sensitivity to CD14+ depletion and sensitivity to ibrutinib or idelalisib (Supplementary Figure 3D-3E), suggesting that the mechanism underlying the loss of cell viability after CD14+ depletion is specific to CSF1R inhibitor sensitivity and not with sensitivity to any effective small-molecule kinase inhibitor.

Based on our hypothesis that CSF1R inhibition is mediated by the CD14-positive cells, we predicted that CD14+ depletion would prevent any further impact of CSF1R inhibitors on the viability of the remaining cells post-depletion. To test this prediction, we examined the correlation between the change in CSF1R sensitivity imparted by CD14+ depletion relative to the sensitivity to CSF1R inhibitors of the whole mononuclear cell fraction (Supplementary Figure 4A). We determined the degree to which CSF1R dose-response curves were altered after CD14+ depletion *versus* the CSF1R dose-response curves of whole mononuclear cells from the same specimens. We observed that specimens with higher CSF1R inhibitor sensitivity in whole mononuclear cells were the same ones that showed the greatest decrease in sensitivity after CD14+ depletion (*p* = 0.04 for GW-2580; *p* = 0.13 for ARRY-382) (Supplementary Figure 4B-4C). We did not observe this same correlation when comparing ibrutinib or idelalisib sensitivity (Supplementary Figure 4D-4E), again supporting the observation that CSF1R sensitivity is directly connected to the CD14-positive cell population.

In chronic lymphocytic leukemia, CD14-positive monocytes can differentiate into nurse-like cells (NLCs) that provide a critical tumor-promotional and -protective microenvironment for the leukemia. To directly confirm that CSF1R inhibition was targeting the subpopulation of supportive CD14-expressing nurse-like cells (NLCs), we performed long-term culturing experiments to isolate adherent NLCs from the bulk CLL cell population [7]. We exposed primary patient specimens to CSF1R inhibitors before culturing NLCs and measured the change in the number of NLCs that grew on the plate. We found that the addition of 1µM GW2580 or ARRY-382 dramatically decreased the number of NLCs compared to untreated cells (Figure 4C). Interestingly, for one patient specimen unable to produce a significant number of NLCs even in the untreated condition, the CSF1R inhibitors did not decrease viability, suggesting that their efficacy is dependent on the presence and activity of NLCs within the leukemia (Figure 4D).

Overall, these results demonstrate that the depletion of CD14-positive cells, or the removal of these cells after their differentiation into nurse-like cells, results in decreased CLL cell viability and decreased sensitivity to CSF1R inhibitors. They demonstrate that the effectiveness of CSF1R inhibitors is dependent on a paracrine interaction of leukemic cells with CD14-positive/CSF1R-positive supportive monocytes.

### CSF1R inhibitors work synergistically in combination with ibrutinib and idelalisib in a majority of CLL patient samples

**Figure 5:**
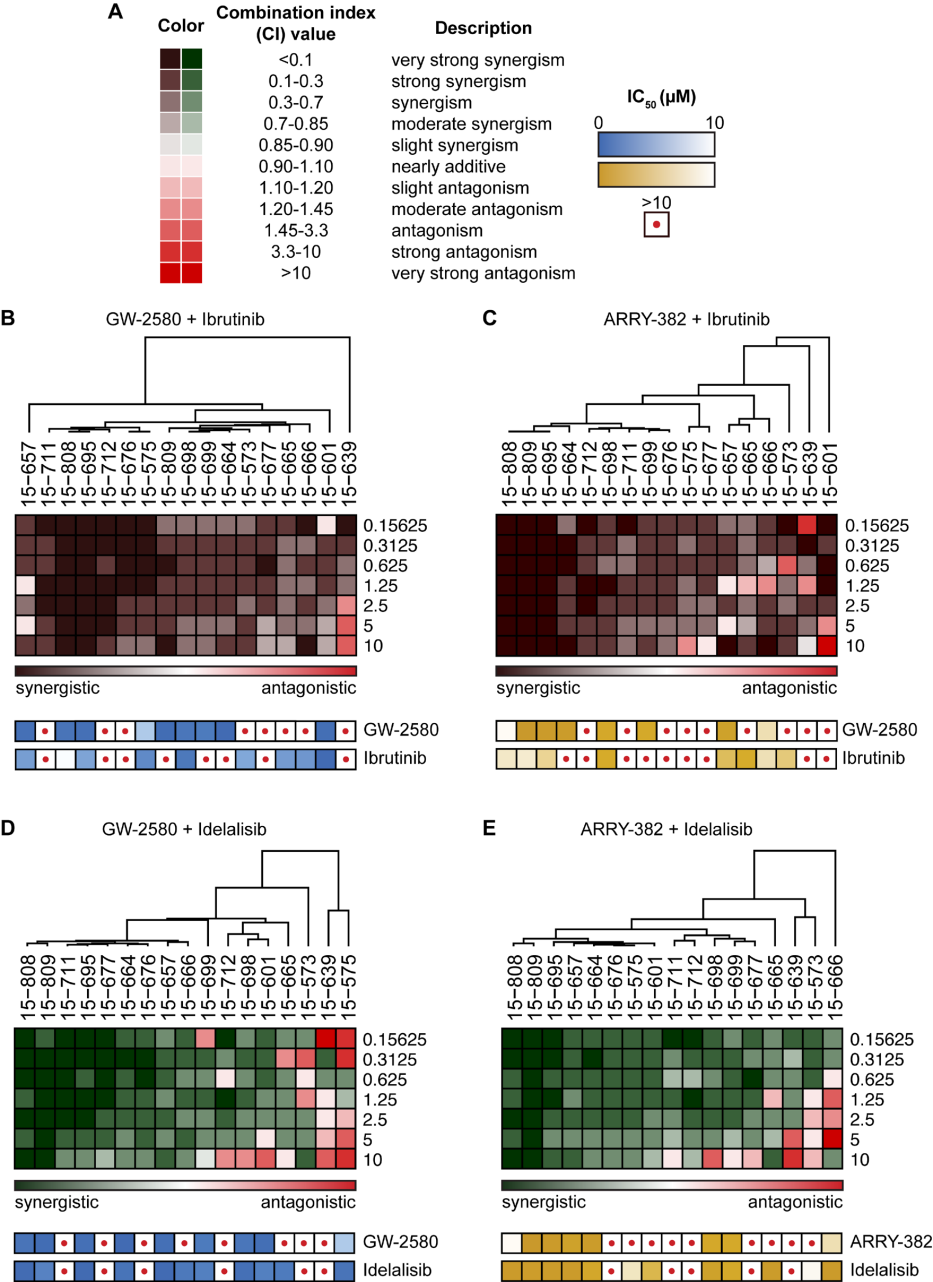
Synergy between ibrutinib or idelalisib and CSF1R inhibitors in majority of CLL patient specimens **A.** Mononuclear cells from CLL patient specimens were cultured with dose gradients of single-agent CSF1R inhibitors, ibrutinib, or idelalisib, as well as equimolar ratio dose gradients of CSF1R inhibitors combined with ibrutinib or idelalisib. After a 72-hour incubation, relative numbers of remaining viable cells were assessed using a colorimetric cell viability assay, and synergy calculations were generated from the dose-response curves. **B.**-**E.** A hierarchically clustered heat map was generated showing the combination indices at increasing concentrations of inhibitors (rows) in CLL patient samples (columns) for **B.** GW-2580 with ibrutinib; **C.** ARRY-382 with ibrutinib; **D.** GW-2580 with idelalisib; and **E.** ARRY-382 with idelalisib. The single-agent sensitivity (IC50) to each drug used in the combination is included (depicted as a heat map) below the corresponding patient sample.

To determine the potential utility of combining CSF1R inhibitors with currently approved therapies for CLL, we evaluated the sensitivity of patient samples to GW-2580 and ARRY-382 in combination with ibrutinib or idelalisib. CLL primary patient samples were exposed to each CSF1R inhibitor alone and to combinations of each inhibitor with ibrutinib or idelalisib in equimolar concentrations, and combination index (CI) values were calculated (Figure 5A) [31].

We observed a strong synergistic effect when combining CSF1R inhibitors with ibrutinib or idelalisib in the majority of patient specimens (Figure 5B-5E; see Supplementary Figure 5 for examples of the drug-dose response curves from representative synergistic and antagonistic patient samples). To assess the possibility that this effect was driven primarily by single-agent sensitivity, we performed unsupervised hierarchal clustering of CI values across the range of drug concentrations, grouping specimens into a spectrum from most to least synergistic. We compared the IC50s of the single agents across the spectrum of patient samples and did not observe any significant association between single-agent sensitivity and combination synergy (Figure 5B-5E). Moreover, many patient samples that had been resistant to ibrutinib and idelalisib alone became sensitive to the inhibitor in combination with GW-2580 or ARRY-382, suggesting the broad applicability of using CSF1R inhibitors with currently approved inhibitors to target CLL.

## DISCUSSION

The implementation of kinase inhibitors in CLL such as ibrutinib and idelalisib has shown durable response in patients with refractory or poor-risk disease [32-34]. However, as quickly as ibrutinib reached success, a population of patients began to develop resistance [35], resulting in significantly decreased overall survival [36]. In our study, we focused on therapeutic approaches that would potentially have a lasting, significant impact in treating CLL patients: successfully targeting both leukemic cells and their protective NLCs.

Many solid tumors depend upon microenvironmental support through TAMs [37, 38], which enhance primary tumor growth and suppress the immune response [39]. There is a similar contribution of the microenvironment in hematologic malignancies, including CLL and lymphoma [8, 40, 41]. For example, LAMs, which highly express CSF1R, have been found to support Hodgkin and non-Hodgkin’s lymphoma [42]. In Hodgkin lymphoma patients treated with standard chemotherapy, higher CSF1R expression was associated with shorter survival [43, 44]. In CLL, the importance of NLCs has been widely demonstrated, and CD14-positive NLC monocytes are critical in maintaining CLL cell viability, and depletion of cells from CLL patient specimens results in leukemic cell death [8, 40].

In this study, we demonstrated a novel mechanism for targeting of the CLL microenvironment using two highly selective small molecule inhibitors of CSF1R. Through *ex vivo* functional screening of 197 CLL patient specimens, we found that more than 25% of these CLL specimens are highly sensitive to CSF1R inhibition (Figure 1). While *ex vivo* screening can result in variability among cell subpopulations from sample to sample, we aimed to reduce the impact of variability by using a large data set. Furthermore, the variability seen likely reflects the inherent biologic differences across patient samples and therefore potentially represents the variety of drug responses that may be observed *in vivo*.

A comparison of CSF1R sensitivity against various clinical, genetic, and cytogenetic characteristics revealed no major correlations. We did observe a trend toward white cell count correlating with CSF1R sensitivity, and flow cytometric analysis revealed a correlation between CSF1R sensitivity and quantitative levels of CD14-positive cells (Figure 3). These interesting correlations are consistent with recently reported clinical data which showed that these characteristics resulted in a shorter time to initiation of treatment and reduced overall survival [12].

Furthermore, we also observed that primary CLL specimens contain a sub-population of CSF1R+/CD14+ cells (Figure 3A), suggesting that CSF1R signaling may be an important marker and novel target of the CLL microenvironment. Consistent with these findings, we have also validated the findings that CD14+ depletion deleteriously impacts on CLL cell viability, and we show for the first time in patient samples using selective small-molecule inhibitors that this phenomenon is mimicked by CSF1R inhibition, suggesting a new potential therapeutic route to target the CLL microenvironment.

A recent theme in clinical oncology research and patient care is the need for combination therapies. The contribution of agents targeting tumor-associated macrophage/monocyte lineage cells in these combination regimens has been robustly demonstrated in solid tumors, in which inhibition of microenvironmental cell types (such as TAMs) dramatically synergizes with tumor-directed therapies *via* inhibition of microenvironmental rescue signals and induction of immune responses against tumor cells [45, 46]. Our findings extend this concept of targeting TAM-promoted neoplasia into hematologic malignancies. In addition, we show that combinations of CSF1R inhibitors with tumor-directed therapies in CLL (ibrutinib or idelalisib) exhibit strong synergy across numerous CLL patient specimens (Figure 5). These data are suggestive of candidate combination therapy regimens for CLL patients that may improve the duration of response and mitigate single-agent drug resistance. Simultaneous targeting of BTK and CSF1R may be of particular interest. Despite the ability of ibrutinib to disrupt CLL cell interactions within their protective niches, inhibiting BTK in NLCs may actually promote CLL cell survival, which may explain the inability of ibrutinib to overcome the protective effects provided by NLCs [47, 48].

A recent report showing that pharmacologic depletion of macrophages in a CLL cell-line mouse model with a liposomal formulation of a bisphosphonate (clodrolip) or an anti-CSF1R monoclonal antibody (emactuzumab) results in significant anti-leukemic activity [22]. In addition, it has been recently shown that CSF1R is expressed in NLCs and in lymph nodes derived from CLL patients, and that neutralization or inhibition of CSF1R inhibits NLC formation, decreases CLL cell viability, and enhances the anti-tumor effects of ibrutinib [24]. This research further supports the potential for targeting CSF1R in CLL.

Ultimately, these results suggest a treatment strategy whereby CSF1R+/CD14+ cells can be chemically targeted by highly selective CSF1R inhibitors, and that this targeting deprives CLL cells of crucial microenvironmental support. We propose enhancing this strategy in CLL patients through combination drug approaches by simultaneously giving CSF1R inhibitors, which target NLCs, with BTK and/or PI3Kδ inhibitors as well as other agents, which target the leukemia cells themselves.

## MATERIALS AND METHODS

### Patient sample acquisition and processing

Primary leukemia samples were obtained from CLL patients by informed consent according to a protocol approved by the Institutional Review Board at Oregon Health & Science University. These samples were subsequently processed and exposed to an *ex vivo* small-molecule inhibitor screen as described previously [49]. Briefly, peripheral blood samples were extracted from CLL patients and the fresh mononuclear cells were isolated from whole blood using a Ficoll density gradient. The isolated cells were plated in R20 media consisting of RPMI (#11875; Thermo Fisher, Waltham, MA) with 20% FBS (#S11550; Atlanta Biologicals, Lawrenceville, GA), 1% penicillin-streptomycin (#15140; Thermo Fisher), 2% glutamine (#25030; Thermo Fisher), and 0.1% amphotericin B (#SV3007801; Thermo Fisher).

The mononuclear cells were plated with dose-escalating concentration gradients of small-molecule inhibitors, or a combination of inhibitors following the same fixed concentrations, and incubated for 72 hours at 37°C in 5% CO_2_. After incubation, the relative number of remaining viable mononuclear cells in the plate was measured using a tetrazolium-based colorimetric assay (CellTiter AQueous One Solution Cell Proliferation Assay; Promega, Madison, WI). To determine the degree of apoptosis after exposure to CSF1R inhibitors, the mononuclear cells were plated with either 1uM or 10uM of GW-2580 (GlaxoSmithKline, Brentford, United Kingdom) or ARRY-382 (Array BioPharma, Boulder, CO). The percentage of apoptotic cells was measured after 24, 48, and 72 hours using the Guava Nexin Assay (Merck Millipore, Billerica, MA)

Dose-response curves were generated for GW-2580 (*n* = 191 specimens) and ARRY-382 (*n* = 131), along with the BTK inhibitor ibrutinib (*n* = 84) (AbbVie, North Chicago, IL) and the PI3Kδ inhibitor idelalisib (*n* = 160) (Gilead Sciences, Seattle, WA). Based on cell availability, multiple replicates for each inhibitor dose-response curves were generated on different test plates using our *ex vivo* screen. To calculate an overall drug sensitivity profile for each sample, for each inhibitor replicate, outliers were manually removed and the IC50 and AUC were calculated after fitting the data using a third-order polynomial regression model. Inhibitor curves were removed from further analysis if: (1) AUC < 1100 and (2) r < 0.4 (Pearson’s correlation coefficient). Additionally, test plates were removed if the percent standard deviation (%stdev) among replicates—calculated as (mean ÷ stdev) * 100—was less than 50%. Similarly, samples were removed if the %stdev among test plates was less than 50%.

Most clinical and genetic information was collected during routine standard-of-care patient sample evaluation obtained using electronic medical records. Some samples that had not been evaluated for the presence of immunoglobulin heavy chain (IGHV) gene mutations during initial treatment were subsequently analyzed using the IGH Somatic Hypermutation Assay v2.0 (#5-101-0031; Invivoscribe, San Diego, CA). The NCBI IgBLAST tool was used to determine the percent divergence of each clonal sequence, where samples with equal or less than 2% divergence from the germline sequence were considered to have non-mutated IGHV.

### Immunophenotype analysis of CLL patient samples

Patient sample mononuclear cells that had undergone Ficoll gradient isolation were immunophenotyped to standard clinical specifications by the OHSU Histopathology Shared Resource laboratory. The following antibodies were used: CD3-FITC (#349201; BD Biosciences, Franklin Lakes, NJ), CD5-PC-Cy7 (#348790; BD Biosciences), CD14-APC-H7 (#643077; BD Biosciences), CD19-V450 (#644492; BD Biosciences), CD33-PerCP-Cy-5.5 (#341640; BD Biosciences), CD45-Pacific-Orange (#MHCD4530; Invitrogen, Carlsbad, CA), CD64-PE (#558592; BD Biosciences), and CSF1R-APC (#347306; BioLegend, San Diego, CA). Surface marker analysis was performed on a BD FACSCanto II flow cytometer and the data were analyzed using FlowJo (FlowJo, LLC, Ashland, OR).

### Depletion of CD14+ cells from primary patient specimens

Primary patient mononuclear cells were depleted of CD14-expressing cells using MACS MicroBead technology (Miltenyi Biotec, Bergisch Gladbach, Germany). CD14-expressing cells were labeled with magnetic anti-CD14 MicroBeads (#130-050-201; Miltenyi Biotec), resuspended in MACS buffer (phosphate-buffered saline pH 7.2, 0.5% bovine serum albumin, and 2 mM EDTA) per protocol, and separated with MACS MS columns (#130-042-201; Miltenyi Biotec). Cell viability of CD14+ depleted and non-depleted mononuclear cells was measured using the Guava easyCyte cell counter (Merck Millipore).

Both CD14+ depleted and non-depleted mononuclear cells from the same specimen were plated in R20 cell culture media with or without a concentration gradient of GW-2580 or ARRY-382. Relative numbers of viable cells were measured at 0, 24, and 72 hours after plating using a tetrazolium-based colorimetric assay.

### Isolating nurse-like cells (NLCs) from primary patient specimens

Long-term NLC culturing experiments were conducted based on established protocols [7]. For each patient specimen, primary patient mononuclear cells were isolated and pipetted into 6 wells of a 24-well plates (1ml at 1.5 x 10^7^ cells/ml in R20 media). Three wells were exposed to inhibitors immediately after plating (1µM GW-2580, 1µM ARRY-382, and untreated). The cells were incubated for 14 days at 37°C in 5% CO_2_, after which the non-adherent cells were removed from all wells by vigorous pipetting and cell viability for each well was calculated using the Guava easyCyte cell counter (Merck Millipore).

For the three pre-treated wells, the remaining adherent NLCs were washed with RPMI media and exposed to 500µl 5mM EDTA in PBS for 30 minutes, followed by 100µl trypsin for 10 minutes. The cells were spun down, resuspended in R20 media, and counted using a hemocytometer. For the remaining three wells, the non-adherent cells were combined, spun down and resuspended in fresh R20 media. They were pipetted into six wells, three into the original wells containing the adherent NLCs and three into fresh wells. Wells from each group were either exposed to 1µM GW-2580, 1µM ARRY-382, or untreated, and incubated for 72 hours at 37°C in 5% CO_2_, after which the relative number of viable cells was determined using a tetrazolium-based colorimetric assay.

### Synergy calculations between CSF1R inhibitors and ibrutinib/idelalisib

CLL primary patient specimens were exposed to GW-2580/ARRY-382 and ibrutinib/idelalisib, either to each inhibitor alone and in combination with one another in equimolar concentrations. The patient sample cells were incubated for 72 hours and assessed for viability using a colorimetric assay. We used CalcuSyn (Biosoft, Cambridge, Great Britain) to calculate the combination index [50], which measures the degree of synergy for each combination, and binned each value according to established categories of synergy and antagonism [31]. The hierarchical clustering of specimens was performed using the GenePattern platform [51].

## SUPPLEMENTARY MATERIALS FIGURES AND TABLES




