# Supplementary material for: Targeting of colony-stimulating factor 1 receptor (CSF1R) in the CLL microenvironment yields antineoplastic activity in primary patient samples
Source: Oncotarget. 2018 May 15;9(37):24576–89. doi: 10.18632/oncotarget.25191 (PMC5973855; doi:10.18632/oncotarget.25191)
Supplement: Supplementary file 1 [file oncotarget-09-24576-s001.pdf]

Targeting of colony-stimulating factor 1 receptor (CSF1R) in the CLL microenvironment yields antineoplastic activity in primary patient samples

SUPPLEMENTARY MATERIAL

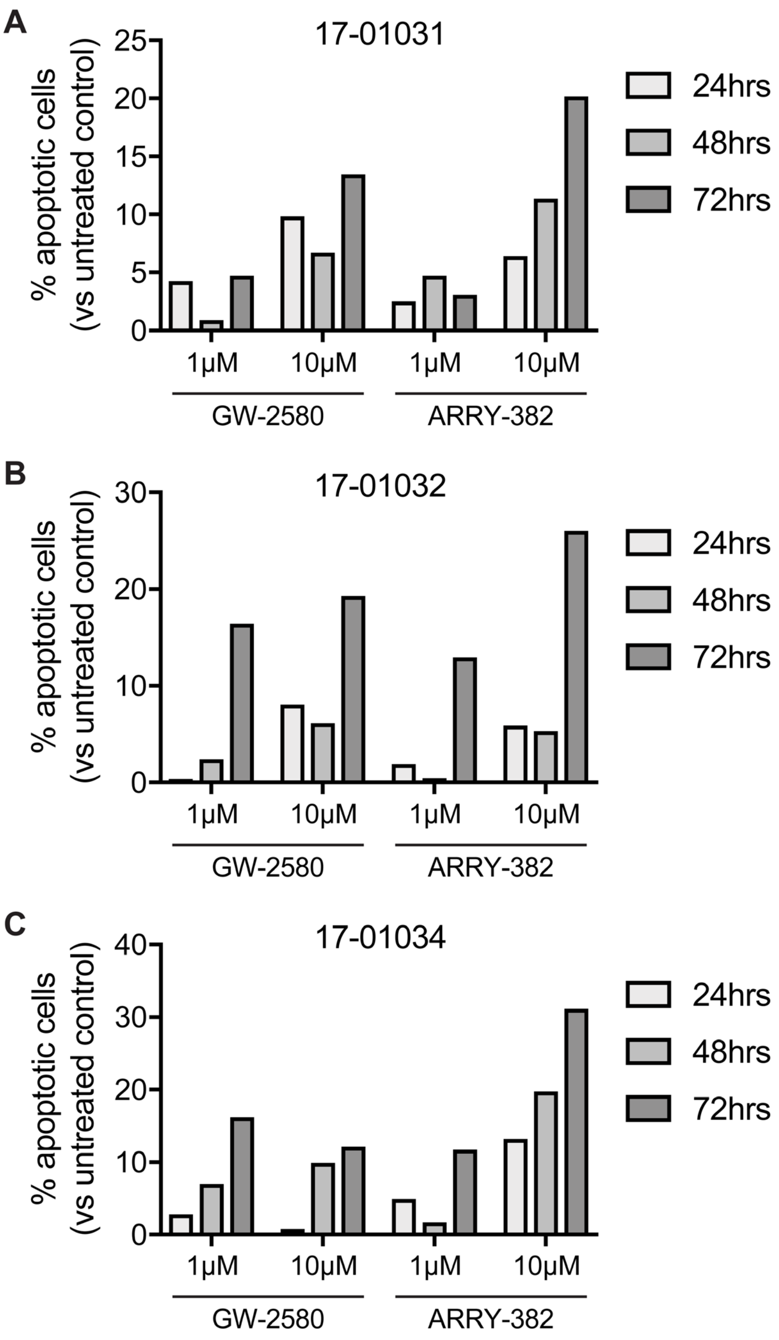

Supplementary Figure 1, related to Figure 1: CSF1R inhibitor exposure induces apoptosis in CLL primary patient samples. A.-C. Percent apoptosis after exposure to GW-2580 and ARRY-382 at 24, 48, and 72 hrs in three CLL primary patient samples: **A.** 17-01031, **B.** 17-01032, and **C.** 17-01034. The percentage of apoptotic cells for each patient sample was normalized to untreated control cells to account for sample-specific variations in cell viability over time.

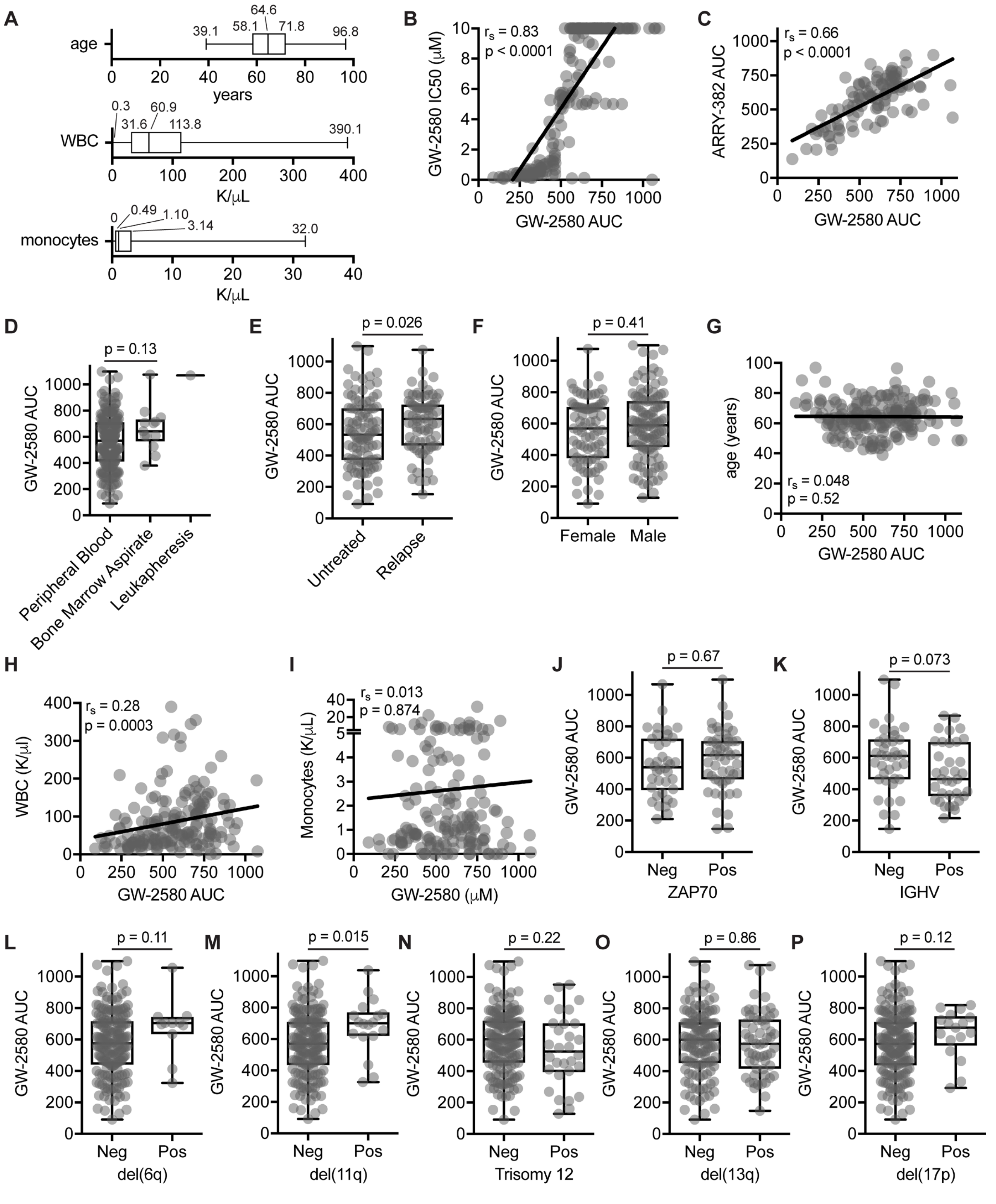

Supplementary Figure 2, related to Figure 2: Box-and-whisker plots for continuous variables in CLL patient sample comparisons and plots of statistically significant characteristics. **A.** Box-and-whisker plots of the clinical characteristics measured by continuous variables that are displayed in Figure 2. **B.-C.** Correlation between GW-2580 area under the curve (AUC) in CLL patient samples and **B.** GW-2580 IC50 and **C.** ARRY-382 AUC. **D.-P.** Association or correlation between GW-2580 AUC and **D.** specimen type; **E.** treatment status; **F.** patient gender; **G.** age; **H.** white blood cell count (WBC); **I.** monocyte count; and the presence/absence of **J.** ZAP70 overexpression, **K.** IGHV mutations, **L.** del(6q), **M.** del(11q), **N.** trisomy 12, **O.** del(13q), and **P.** del(17p). Statistics for **D.-F.**; **J.-P.** was evaluated using Mann-Whitney U test; statistics for **G.-I.** were evaluated using Spearman's rank correlation.

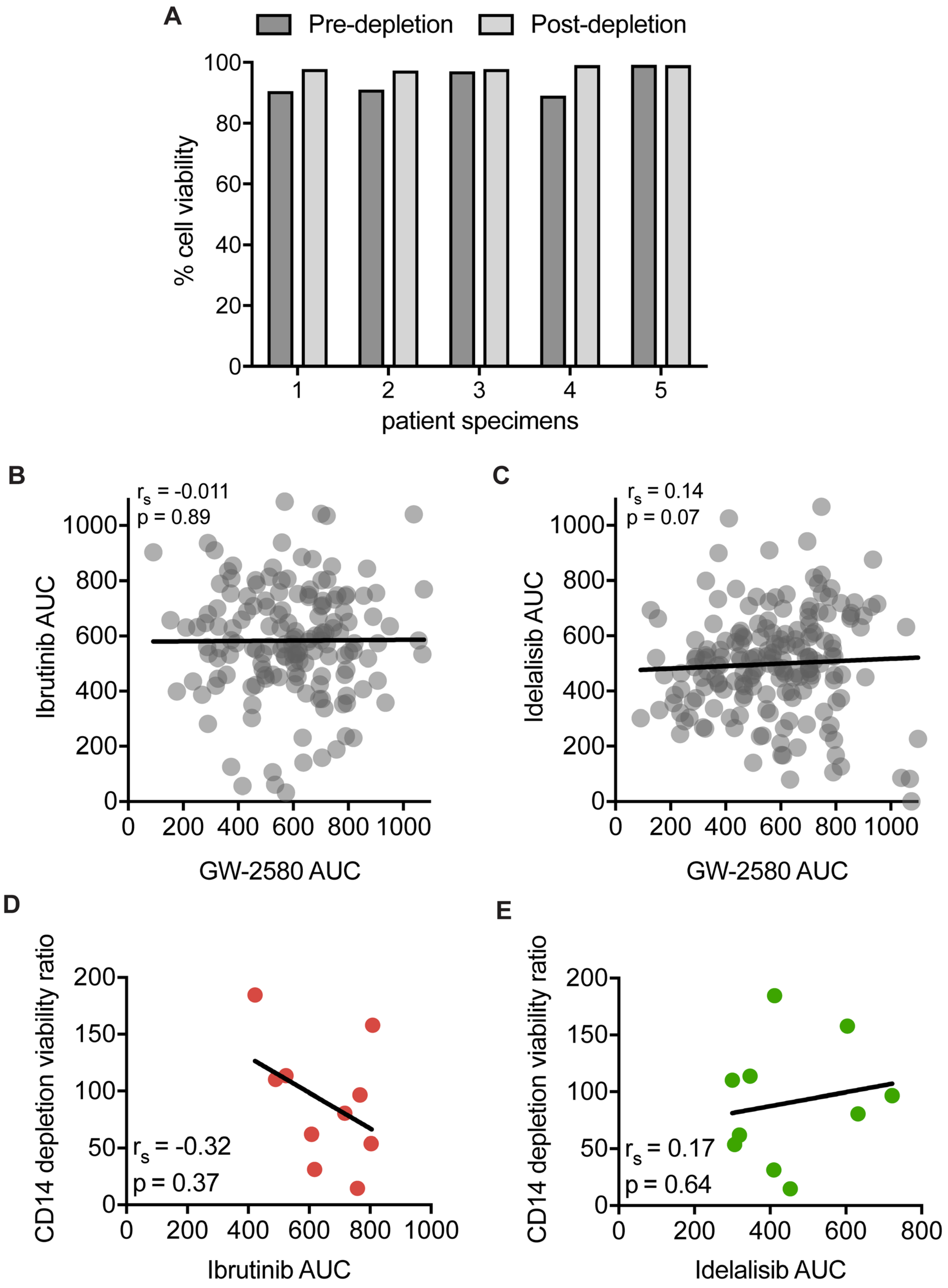

Supplementary Figure 3, related to Figure 4: GW-2580 sensitivity and viability after CD14+ depletion is not correlated with sensitivity to ibrutinib and idelalisib. A. No significant difference in overall cell viability between CLL patient specimens before and after depletion protocol, as measured by Guava easyCyte cell counter. **B.-C.** Correlation between GW-2580 area under the curve (AUC) in CLL patient samples and the AUC values for **A.** ibrutinib and **B.** idelalisib. **D.-E.** CD14+ depletion viability ratio compared to area under the curve (AUC) for **A.** ibrutinib and **B.** idelalisib. Statistics for **B.-E.** determined by Spearman's rank correlation.

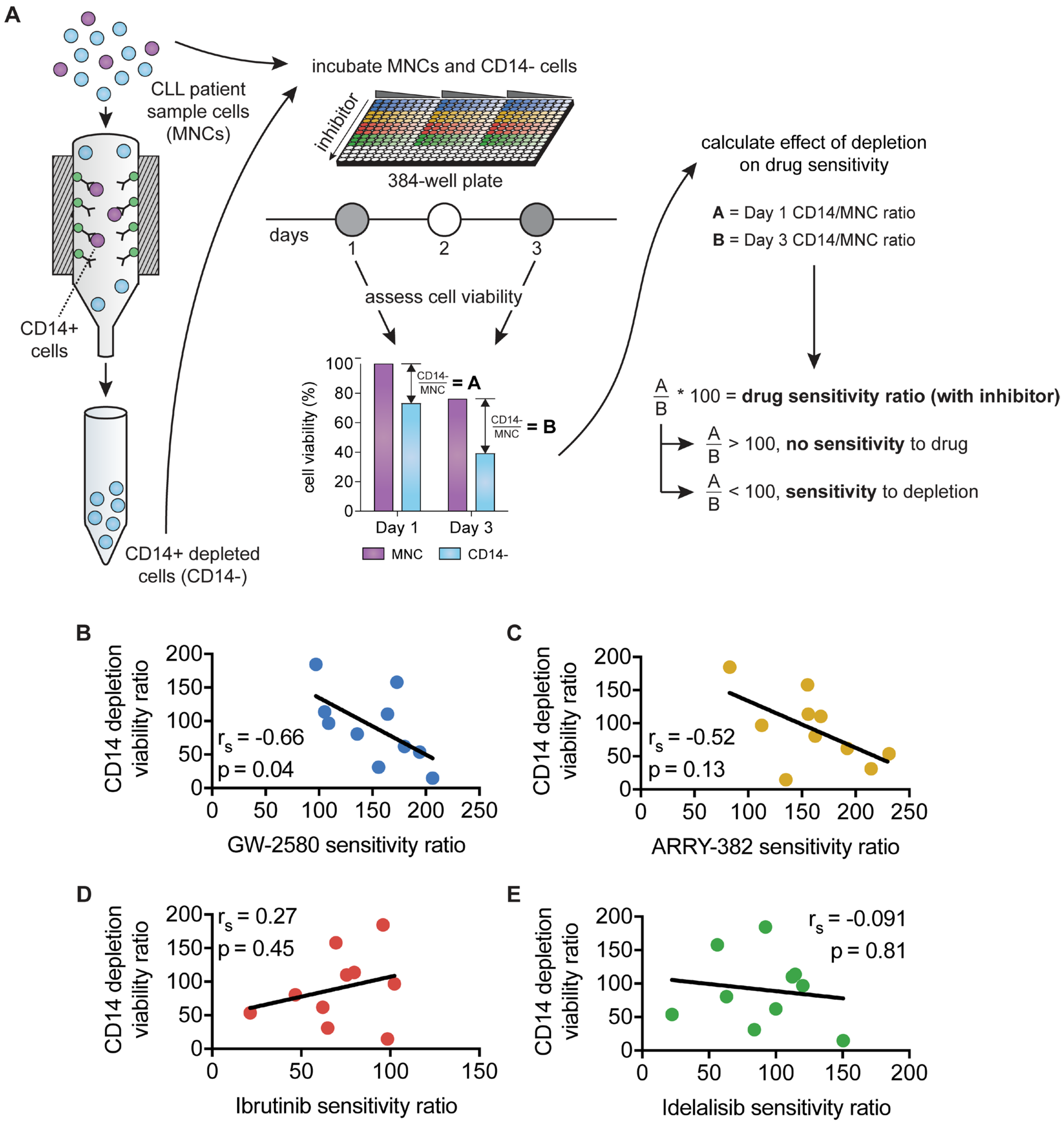

Supplementary Figure 4, related to Figure 4: CD14+ depletion significantly impact sensitivity to CSF1R inhibitors but does not impact sensitivity to ibrutinib and idelalisib. A. CD14+ depleted and whole mononuclear cells were plated with dose-escalating concentrations of inhibitors and incubated for 72 hours. The drug sensitivity ratio was calculated by comparing sensitivity to inhibitors of CD14+ depleted versus whole mononuclear cells.**B.-E.** There is a correlation or trend between the drug sensitivity ratio and the cell viability ratio for CD14+ cell depletion after exposure to CSF1R inhibitors—**B.** GW-2580 and **C.** ARRY-382—but not to **D.** ibrutinib and **E.** idelalisib. Statistics determined by Spearman's rank correlation.

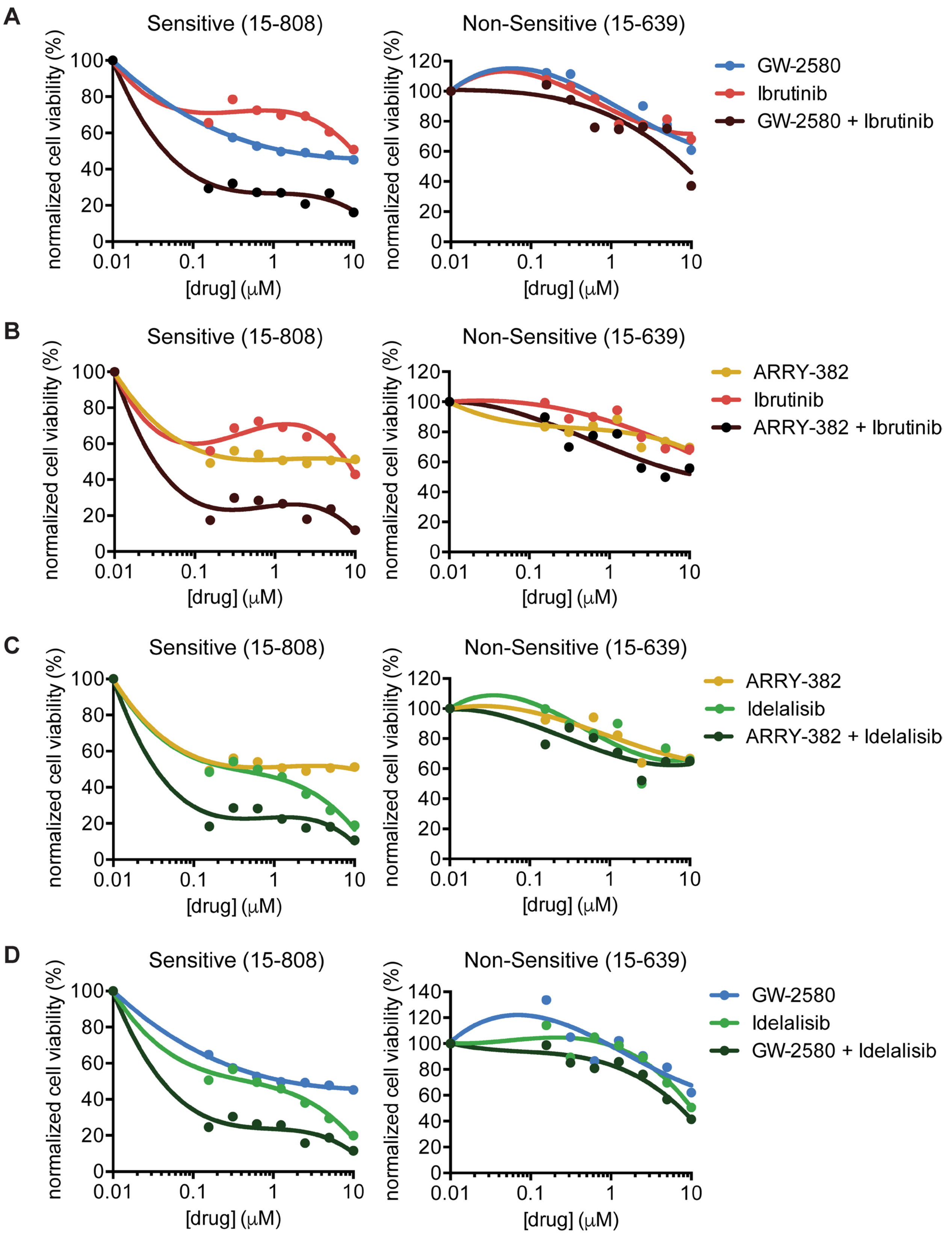

Supplementary Figure 5, related to Figure 5: Representative synergistic and antagonistic dose-response curves from patient specimens exposed to the combination of CSF1R inhibitor and ibrutinib/idelalisib. Dose-response curves for each single agent inhibitor and combination for **A.** GW-2580 and ibrutinib, **B.** ARRY-382 and ibrutinib, **C.** ARRY-382 and idelalisib, and **D.** GW-2580 and idelalisib in which the combination was synergistic (Specimen 15-808) and non-synergistic (Specimen 15-639).

Supplementary Table 1, related to Figure 2: Complete table of clinical and genetic/cytogenetic characteristics for 197 primary CLL patient samples evaluated for sensitivity to CSF1R inhibitors. "NA" = not available.

For Supplementary Table 1 see attached excel file in Supplementary Files.

Supplementary Table 2, related to Figure 2: Statistical analysis of clinical and genetic/cytogenetic characteristics of CLL patient sample cohort screened for CSF1R inhibitor sensitivity.

Category	Samples with available data (%)	Statistical test	Correlation coefficient	*P*-value
Inhibitor sensitivity				
GW-2580 IC50	197 (100)	Spearman's rank correlation	0.8291	<0.0001
ARRY-382 IC50	102 (52)	Spearman's rank correlation	0.5790	<0.0001
Clinical				
specimen type	196 (100)	Mann-Whitney test	--	0.1301
treatment status	177 (90)	Mann-Whitney test	--	0.0259
gender	193 (98)	Mann-Whitney test	--	0.4080
age	185 (94)	Spearman's rank correlation	0.0484	0.5128
WBC (K/µL)	171 (87)	Spearman's rank correlation	0.2761	0.0003
monocytes (K/µl)	159 (81)	Spearman's rank correlation	0.0127	0.8740
Mutations				
ZAP70	89 (45)	Mann-Whitney test	--	0.6680
IGVH	73 (37)	Mann-Whitney test	--	0.0726
Cytogenetics				
del(6q)	191 (97)	Mann-Whitney test	--	0.1094
del(11q)	191 (97)	Mann-Whitney test	--	0.0154
trisomy 12	191 (97)	Mann-Whitney test	--	0.2151
del(13q)	191 (97)	Mann-Whitney test	--	0.8621
del(17p)	191 (97)	Mann-Whitney test	--	0.1237
